# G-Quadruplex-Forming Aptamers—Characteristics, Applications, and Perspectives

**DOI:** 10.3390/molecules24203781

**Published:** 2019-10-21

**Authors:** Carolina Roxo, Weronika Kotkowiak, Anna Pasternak

**Affiliations:** Department of Nucleic Acids Bioengineering, Institute of Bioorganic Chemistry, Polish Academy of Sciences, Noskowskiego 12/14, 61-704 Poznan, Poland

**Keywords:** aptamers, G-quadruplexes, cancer, anticoagulants, aptasensors, antiviral agents, therapeutics, diagnostics, conjugates

## Abstract

G-quadruplexes constitute a unique class of nucleic acid structures formed by G-rich oligonucleotides of DNA- or RNA-type. Depending on their chemical nature, loops length, and localization in the sequence or structure molecularity, G-quadruplexes are highly polymorphic structures showing various folding topologies. They may be formed in the human genome where they are believed to play a pivotal role in the regulation of multiple biological processes such as replication, transcription, and translation. Thus, natural G-quadruplex structures became prospective targets for disease treatment. The fast development of systematic evolution of ligands by exponential enrichment (SELEX) technologies provided a number of G-rich aptamers revealing the potential of G-quadruplex structures as a promising molecular tool targeted toward various biologically important ligands. Because of their high stability, increased cellular uptake, ease of chemical modification, minor production costs, and convenient storage, G-rich aptamers became interesting therapeutic and diagnostic alternatives to antibodies. In this review, we describe the recent advances in the development of G-quadruplex based aptamers by focusing on the therapeutic and diagnostic potential of this exceptional class of nucleic acid structures.

## 1. Introduction

Aptamers are small DNA- or RNA-based oligonucleotides which are typically produced by the systematic evolution of ligands by exponential enrichment (SELEX) technology. The term aptamer is derived from the Latin word “*aptus*,” meaning “to fit,” and the Greek word “*meros*,” meaning “part.” Under certain conditions, aptamers can fold into three-dimensional structures. Structural motifs within aptamers provide specific binding sites for small molecules or macromolecular compounds of several types, including cells, cell surface proteins, bacteria, and viruses; moreover, they interact with targets with high affinity and selectivity [1,2]. Aptamers are called chemical antibodies, but they have huge advantages over them, like increased stability, less expensive and less time-consuming production, ease of chemical modification, lower immunogenicity, and higher target range.

Aptamers have evolved from SELEX technology. The acronym SELEX refers to the technique independently developed around 1990 by groups of Gold and Szostak [3]. SELEX is the process by which aptamers are discovered and begins with the construction of a randomly generated library. Each sequence is unique and contains random bases (20–50 nt) flanked by two conserved primer binding sites, which are used for PCR amplification by hybridizing primers. The conventional SELEX method generally involves three steps: selection, partitioning, and amplification. In the selection step, the library is incubated with target molecules for the indicated time. After incubation, the unbound sequences are separated from those that are bound. In DNA SELEX, the target-bound sequences are amplified by PCR; however, in RNA SELEX, reverse transcription PCR must be applied. After several selection rounds, the enriched pool of oligonucleotides is sequenced, and their binding abilities are further evaluated [4].

The selection of specific candidates for aptamers is time-consuming. Thus, several modified SELEX methods have been set up to decrease the selection time and enhance the hit rates. One of such method is *negative* SELEX, where after three selection cycles, the library is incubated with chromatography columns as negative selection and the non-specific binding sequences are then removed from each pool [4]. In another approach, named *counter* SELEX, an extra step, using structurally similar targets, is introduced for incubation with aptamers to effectively discriminate non-specific oligonucleotides [4]. An interesting approach is taken in *capillary electrophoresis* SELEX, where the target-bound sequences and unbound sequences are separated by the difference in electrophoretic mobility, which is a highly efficient separation method [5]. A modified selection technology is microfluidic SELEX, which merges traditional SELEX with a microfluidic system [6]. Recently, there has been growing interest in the application of whole live cells as targets (*cell* SELEX), which increases the possibility of the selected aptamer being used for diagnostic and therapeutic applications [7]. Moreover, a novel in vivo selection process, named in vivo SELEX, was designed to generate RNA motifs capable of localizing to intrahepatic tumor deposits [8]. An excellent tool for recognizing the best aptamers for targets seems to be *high-throughput sequencing* SELEX. In this method, the library is sequenced across all the selection rounds. Consequently, enriched sequences are visible in much earlier rounds which makes the selection process more time-efficient, but more expensive [9]. In 2017, Albanese et al. described a genome-inspired reverse selection method to overcome the limitations of SELEX technology [10]. This method uses specific DNA sequences from the human genome to capture proteins, taking advantage of the eons of biological evolution of DNA sequences that selectively interact with proteins to perform biological functions. Linking aptamer discovery to the sciences field increases the rate of discovery of highly specific protein-DNA interactions that have biological significance and analytical utility. Although various SELEX protocols have been developed, only a few aptamers entered clinical trials, while some presented very promising properties to be examined. The high specificity and efficiency of the selection process is still the most challenging limitation.

G-rich oligomers comprise a large group of aptamers with the ability to fold into stable G-quadruplex (G4) structures under physiological conditions and recognize different proteins [11,12]. G4 are non-canonical nucleic acid structures stabilized by the stacking interactions of G-quartets, in which four guanines are assembled in a planar arrangement by Hoogsteen hydrogen bonding [2,13]. The structure of G4 is widely polymorphic, which indicates that it can be formed by one, two, or four separate strands of DNA or RNA [14]. Moreover, the strands directions can have various combinations and the arrangement of the G-quartets/strands can be parallel, antiparallel, or hybrid. Moreover, they differ in loops size and sequence.

G-rich aptamers that form G4 have several advantages compared with unstructured sequences [2]. They are thermodynamically and chemically stable, show no immunogenicity and are resistant to numerous serum nucleases. It is noteworthy, that G4 are characterized by enhanced cellular uptake [15,16]. The stability of G4 structure is very important for improving electrostatic interactions with the positively charged binding ligands because its structure has twice negatively charged density per unit length compared to the duplex DNA.

In the past few years, a number of G-rich aptamers have been developed and their potential has been used in many ways such as anticoagulants, [17] therapeutic agents for cancer therapy [18], for treatment of other diseases [19], and as nano-devices [20] and aptasensors [21].

In this review, we summarize the recent developments and applications of G-rich aptamers in therapy, drug delivery, and diagnostics.

## 2. Anticoagulant Agents

Blood coagulation process is a complex, tightly connected cascade of reactions, in which the product of one reaction participates in the generation of the next one [22]. The main goal of this process is to convert serum-soluble fibrinogen into insoluble fibrin, which is the substantial component of blood clot [23]. The formation of stable hemostatic plug is crucial for preventing blood from leaking out from blood vessels and maintaining hemostasis in the organism. The disruption of the blood coagulation cascade is observed in cancer therapy, orthopedic injuries, and cardiovascular diseases. The understanding of the process lets researchers create various anticoagulant compounds such as warfarin, low-molecular-weight heparin, and dabigatran. Notably, nucleic acids aptamers constitute a unique class among them.

**Figure 1:**
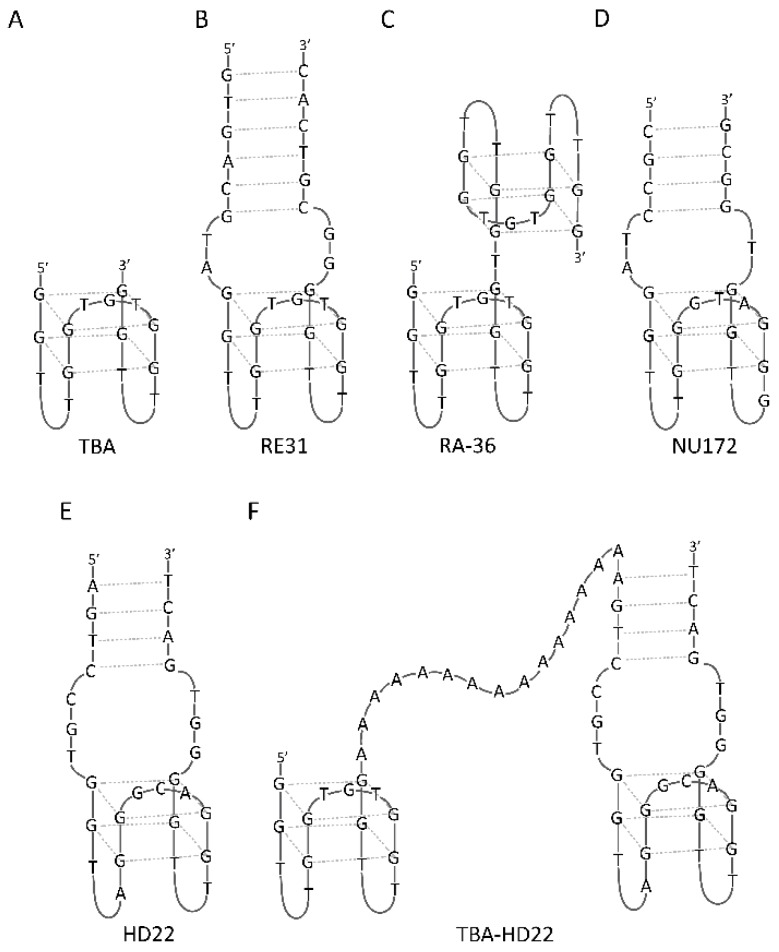
G-quadruplex models for some anticoagulant agents.

The first and the most-well known anticoagulant aptamer was the thrombin binding aptamer (TBA, also termed HD1, ARC183), discovered by Bock in 1992 using SELEX [24]. The TBA sequence comprises 15 deoxynucleotide residues (Figure 1A) and adopts an intramolecular, antiparallel G4 structure with chair-like conformation [25]. The core of TBA is formed by two guanosine quartets, connected by two shorter TT loops and one longer TGT loop. The aptamer structure is stabilized by a potassium ion [25] located in the G4 core [26]. Crystallographic and NMR studies show that TBA exerts its anticoagulant properties by binding to thrombin with TT loops which act as a pincer-like system that embraces the protruding fragment of the thrombin exosite I [27,28]. The first in vivo experiments conducted on cynomolgus monkeys and dogs showed that TBA was able to prolong plasma prothrombin time in a linear dose-response manner [29,30]. Further studies revealed unique among anticoagulants abilities of TBA to inhibit clot-bound thrombin activity and platelet aggregation [31]. The results of the in vivo experiments were promising enough to let Archemix Corporation initiate clinical trials in which TBA was evaluated as an anticoagulant for coronary artery bypass graft surgery [32]. Unfortunately, TBA exhibited a suboptimal dosing profile; therefore, more advanced tests were suspended [33,34]. However, favorable TBA properties such as reversibility of action, small size, lack of side effects, and simplicity of chemical synthesis elicited further studies. A wide variety of chemical modifications have been used to improve TBA affinity to thrombin or prolong its thermodynamic and biological stability. Enhancement of TBA anticoagulant properties was observed for TBA variants modified with the introduction of 2′-deoxy-2-alkylguanosine [35], 2′-deoxy-8-alkylguanosine [35], 2′-deoxy-d-/l-isothymidine [36], 2′-deoxy-4-thiouridine [37], 2′-deoxy-5-fluorouridine [38], unlocked nucleic acids [39,40], formacetal linkage [41], 5-hydroxymethyl-2′-deoxyuridine [42], and a C3 spacer [43]. Furthermore, the presence of 2′-deoxy-8-bromoguanosine [44], 2′-deoxy-isoguanosine [45], and 2′-deoxy-2′-fluoro-d-arabinonucleoside [46] is analyzed to see if the aptamer had beneficial influence on its affinity to thrombin. Unfortunately, in the mentioned cases, reports concerning measurements of anticoagulant effect are not available. It is noteworthy that the most significant improvement in TBA anticoagulant properties and affinity toward thrombin was achieved via the introduction of modified nucleoside residues, predominantly at the T7 position. The above could be a result of specific thymidine orientation, which is turned toward the solution and therefore does not interact with the rest of the G4 structure [47,48]. Hence, the substitution of T7 does not perturb any G4-stabilizing interactions and has favorable influence on TBA inhibitory activity.

An equally important feature, which facilitates the determination of the aptamer utility in the treatment of human diseases, is G4 thermodynamic stability. Improvement in this area was possible by the introduction of 2′-deoxy-8-bromoguanosine [44], 2′-deoxy-5-fluorouridine [38], 2′-deoxy-2′-fluoro-D-arabinonucleoside [46], unlocked nucleic acid monomer [39,40], 4-thiouridine [40], 2′,3′-seco-4-thiouridine [40], 2′-C-piperazino-unlocked nucleic acid monomer [49], 5′-5′ inversion of polarity sites [50,51], 5-bromo-2′-deoxyuridine [52], a C3 spacer [43], and anomeric α-nucleotides [53]. The most significant inconvenience of the therapeutic use of TBA is its high susceptibility to nuclease degradation in a biological environment. This problem can be solved inter alia by the inversion of polarity sites at the terminal sequence [54], covalent ligation of the 5′ and 3′ ends of TBA [55], or the introduction of anomeric α-nucleotides [53].

The constant need for the development of more efficient anticoagulant aptamers, characterized by high affinity for the target protein and specificity of action, resulted in attempts to create longer oligonucleotide constructs with a core based on the TBA sequence. The addition of several nucleoside residues, forming a duplex at the ends of the TBA molecule, had a beneficial influence on its biological activity; however, the effect was only visible when at least four nucleotide pairs were introduced [56]. In 2011, Mazurov et al. created a 31-nucleotide DNA aptamer called RE31 (Figure 1B) [57]. The oligonucleotide is formed by the G4 and a duplex domain linked together by four nucleotides in total. The G4 part is a 15-nt fragment with sequence and structure corresponding to TBA, while the duplex domain comprises six pairs of complementary nucleotides. RE31 is characterized by several times lower value of the Kd parameter [58] and increased ability to inhibit thrombin-stimulated clotting reactions and platelet aggregation compared to TBA [56]. Crystallographic data shows that only the G4 region of RE31 is bound to the thrombin exosite [59]. The simultaneous attachment of both aptamer regions to the thrombin molecule is restrained by the adoption of a rod-like shape by RE31. In this structural model, the duplex part of RE31 progressively passes into the G4 structure and the helical axes of the two regions are oriented in the same direction. Nevertheless, additional contacts with the symmetry-related thrombin molecules could be provided by the duplex part, resulting in increased RE31 affinity for its target protein compared to TBA [56,58]. Only a few attempts were made to increase the therapeutic and pharmacokinetic properties of RE31. The LNA and UNA residues turned out to have a beneficial influence on RE31 thermal and biological stability with preservation or improvement of its anticoagulant properties [60]. Furthermore, the prolongation of the inhibitory activity of RE31 was possible because of the preparation of DNA-protamine complexes [61].

RA-36 is another example of an aptamer based on the TBA sequence [62]. This 31-nt long oligonucleotide (Figure 1C) comprises two covalently linked G4 structures: the first one ensures antithrombotic activity, whereas the second one is an aptamer modulator element [63]. Based on experimental data it is known that RA-36 exerts its anticoagulant effect by binding to thrombin exosite I and restraining fibrinogen binding to the enzyme [64,65]. Moreover, it was observed that the full aptamer activity required the presence of potassium ions, which indirectly indicated that the G4 structure is mandatory for achieving RA-36-mediated thrombin inhibition [62]. Furthermore, a study describing in vivo antithrombotic activity of RA-36 in the murine model revealed that the anticoagulant effect of the aptamer was similar to that of bivalirudin, a commercially available peptide drug [64]. The above mentioned findings have led to the designing of other dimeric TBA variants with inversion of polarity and various linkers [66]. Unfortunately, in this case, the direct comparison of their anticoagulant properties with parental RA-36 was not performed; however, evaluation of prothrombin time indicates improved parameters at 2 µM concentration compared to TBA, whereas collapsing of their biological activity at 20 µM oligonucleotide concentration occurs.

One of the most potent anticoagulant aptamers is NU172 (Figure 1D) [67]. The 26-nt long DNA oligonucleotide folds into a mixed duplex/G4 structure. The nucleotide residues of the duplex and G4 region stack continuously provide intramolecular compactness of the NU172 architecture, which ensures a boundary surface for interaction with thrombin exosite I. Consequently, the aptamer is relatively insensitive to the replacement of potassium ions with sodium ions. On the other side, alterations in the aptamer loops provide a shift from an antiparallel to parallel G4 structure while barium and strontium ions are bound [68]. It is noteworthy that only the antiparallel G4 conformation, coordinated with potassium or to a lesser extent with sodium ions, is the functionally active form. Similarly to HD1, NU172 was able to bind to prothrombin and thrombin [69]. Because of the superior anticoagulant properties of the aptamer with favorable pharmacokinetics, clinical trials related to the application of NU172 in heart disease treatments were conducted. G4 was the only thrombin-binding aptamer evaluated in Phase II clinical trials (ClinicalTrials.gov identifier NCT00808964) by ARCA Biopharma [67,70].

The HD22 aptamer (Figure 1E) is a rare example of G4 which inhibits thrombin activity by binding with its exosite II [17]. This DNA aptamer comprises 29 nucleotide residues and folds into a mixed duplex/G4 structure. HD22 is thought to prolong the clotting times by blocking thrombin-mediated activation of factor V [71] and inhibit non-catalytic fibrin polymerization [72]. This aptamer is characterized by high specificity toward thrombin molecule with minimal affinity to prothrombin [69]. Therefore, HD22 was assigned as the most promising candidate among high-performance aptasensors developed for thrombin detection in plasma. Interestingly, efforts to improve the HD22 properties were made by developing hybrid aptamers built with TBA and HD22 linked by poly-A sequence (Figure 1F) [33,73]. The hybrid compound was designed to interact with both thrombin exosites; thus, it was a more effective anticoagulant than each aptamer separately.

## 3. Anticancer Agents

**Figure 2:**
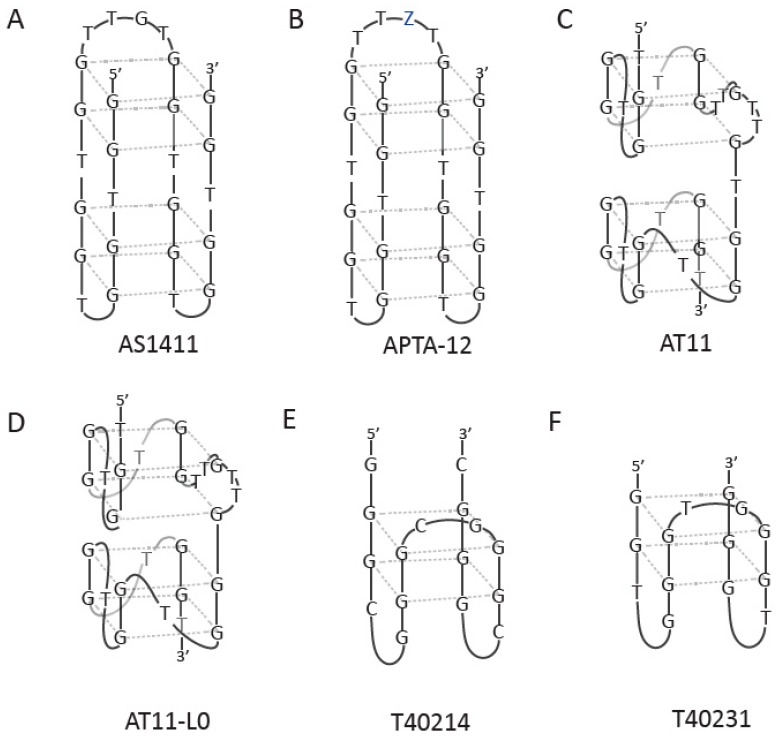
G-quadruplex models of some antiproliferative agents. (**A**) shows one of possible structures formed by AS1411; (**B**) APTA-12 aptamer, Z corresponds to gemcitabine; (**C**) AT11 aptamer; (**D**) AT11-L0 aptamer; (**E**) T40214 aptamer; (**F**) T40231 aptamer.

DNA aptamers demonstrated a great potential to be used in medicine, specifically in cancer therapy. One of the most studied and promising aptamers is the AS1411 aptamer, one of the first aptamers subjected to clinical trials for cancer therapy [74,75]. AS1411 is a 26-mer G-rich DNA oligonucleotide aptamer, which under certain conditions forms an antiparallel G4 structure with intramolecular interaction pattern (Figure 2A). However, its sequence has a high degree of structural polymorphism and, depending on the solution composition and intermolecular interactions, the structure can be changed. This aptamer is highly stable thermodynamically, resistant to nuclease degradation, and has shown promising clinical use. Recently, AS1411, which specifically binds to nucleolin, has been widely and successfully used as a tumor-targeting agent [75]. Nucleolin is a multifunctional, 110-kDa phosphoprotein located primarily in the nucleolus of proliferating cells [76,77]. This protein is related to cell survival, growth, and proliferation as it is overexpressed on the membrane of cancer cells. AS1411 is able to recognize the external domain of nucleolin, thus forming a complex, capable of inhibiting DNA replication and arresting the cells in the S phase of the cell cycle and causing cytotoxicity in cancer cells. Reyes-Reyes et al. suggested that AS1411 was internalized by macropinocytosis via a nucleolin-dependent mechanism [78]. This leads to the sustained activation of Rac1 and causes methuosis, a novel type of non-apoptotic cell death characterized by the hyperstimulation of micropinocytosis. Nevertheless, results of clinical tests with AS1411, as a promising anticancer agent, indicated that this aptamer was safe but had a very rapid human body clearance [79]. The Antisoma company was responsible for the clinical tests, performing the Phase II clinical tests for AS1411 as a monotherapy in patients with renal carcinoma, who failed or were not able to tolerate standard therapy in patients with acute myeloid leukemia [80]. Subsequently, the development of the aptamer as a drug was completed in 2011. Advanced Cancer Therapeutics have acquired the rights to AS1411 and have renamed the compound to ACT-GRO-777.

AS1411 has demonstrated antiproliferative activity in many types of cell lines such as breast, lung, and prostate cancer cell lines. Cheng et al. reported that the AS1411 aptamer induced cell apoptosis, cycle arrest, and decreased the viability of human glioma cells [76]. These events occur by the up-regulation of p53 with the simultaneous down-regulation of Bcl-2 and Akt1 [81]. Moreover, the aptamer inhibited the growth of mice glioma xenograft and prolonged the survival time of glioma tumor-bearing mice. This result suggests a positive development in AS1411 for treating glioma patients in the clinic. Moreover, AS1411 can be applied to different systems, and not only as a drug but also as a transporter, including conjugation to organic and inorganic nanostructures.

One of the examples of nanoparticle conjugates is the AS1411 aptamer conjugated to gold nanoclusters (AS1411–GNCs) [82]. This complex was introduced as a novel targeted radiosensitizer for enhancing the efficacy of radiation therapy, which offers many advantages such as ultra-small size, better cellular uptake, selective targeting of breast cancer cells, and antiproliferative effect [18]. Moreover, AS1411-GNCs were applied along with routine clinical megavoltage radiation for 4T1 breast cancer (a murine tumor model of human breast cancer) to have more translatable results for clinical use. The Apt–GNCs were shown to be useful at cellular uptake, tumor targeting, and enhancement of RT efficacy. Recently, the conjugation of the AS1411 aptamer with a miRNA precursor from human lethal-7 (let-7-d) was studied to target gastric cancer cells with overexpression of nucleolin on their cell surface. The miRNA let-7d is a non-coding RNA and a member of the let-7 family. These family members are reported as tumor suppressors, being let-7d down-regulated in some cancers. Based on this study, it was confirmed that the overexpression of let-7-d by the specific aptamer-mediated delivery system decreases cell proliferation in this cell line. Because of the great potential of the AS1411 aptamer and to meet the constant requirement for improvement and more extensive applications, several modified compounds have been made based on the structure of this aptamer. The APTA 12 aptamer has a mutation with the gemcitabine-incorporated base (Figure 2B), targeting pancreatic cancer cells and delivering gemcitabine (a first-line chemotherapy agent) [83]. Such a construct demonstrated a higher therapeutic effect than gemcitabine *in vivo*. Interestingly, a single internal base mutation and addition of dT at both ends of AS1411 resulted in the formation of a single major G4 conformation with four layers containing two propeller-type, parallel-stranded subunits connected through a central linker (Figure 2C) [84]. This aptamer, named AT11, exhibited a similar antiproliferative activity as AS1411; however, additional studies are required to understand how this G4 may contribute to the cellular uptake and antiproliferative activity. Modification of AT11 by removing one thymine residue between the two G4 subunits leads to various sequence of the AT11-L0 aptamer, which forms a parallel-stranded G4 containing four G-quarters (Figure 2D) [85]. The conjugates of this aptamer with C3, C5, and C8 acridine derivatives demonstrated higher thermal stability than AT11 and AT11-L0. The results suggest that these complexes are a promising delivery system for cytotoxic ligands for cervical cancer treatment. The AS1411 unmodified aptamer and two of its derivatives containing LNA and uracil nucleotides (LNA-AS1411 and U-AS1411) were used as drug delivery systems to carry an acridine orange-based ligand (C_8_) to HeLa cervical cancer cells [86]. From the three aptamers that were tested, the AS1411-C_8_ complex was able to downregulate c-MYC expression; moreover, the uptake of AS1411 was increased in the presence of C_8_. This AS1411-based delivery system can be used for the purpose of accumulation of G4 ligands in the target tissues using a simple non-covalent approach.

Although AS1411 is the most promising and well-known antiproliferative aptamer, other G-rich aptamers have been developed in the last few years for use in anticancer therapy. The T40214 and its analog T40231 aptamers form an intramolecular G4 structure [87]; however, minor differences could be observed between their structures [88]. The T40214 aptamer is 2nt longer than T40231 and has two G-quartets in the center and three C-G loop domains: two at the bottom and one at the top (Figure 2E); however, T40231 has two G-quartets with three T-G loops: two at the bottom and one at the top (Figure 2F). These aptamers significantly suppress the growth of human non-small cell lung cancer (NSCLC) tumors in vivo by selectively inhibiting the activation of Stat3 and its downstream proteins such as Bcl-2, Bcl-xL, Mcl-1, survivin, VEGF, Cyclin D1, and c-myc. The Stat3 protein is a signal transducer and activator of transcription and is involved in cellular differentiation, proliferation, and apoptosis. These results demonstrate that Stat3 is an important molecular target for NSCLC therapy [89]. As a consequence of the specific action of T40214 and T40231, apoptosis is simultaneously promoted with reduction in angiogenesis and cell proliferation. Another G-rich aptamer, named HJ24, has multiple stretches of guanines and forms a parallel G4 structure via intramolecular association [90]. This aptamer recognizes the Shp2 protein and was demonstrated to be an effective inhibitor of Shp2 phosphatase. Shp2 is a member of the protein tyrosine phosphatase (PTP) family which regulates various cellular processes such as cell growth, differentiation, mitotic cycle, and oncogenic transformation. The G4 structure of the aptamer was showed to be crucial for binding to its target; therefore, the HJ24 aptamer action can be reversed using the complementary DNA (cDNA). Application of cDNA can result in disruption of the HJ24 secondary structure and neutralize the inhibitory function of the aptamer. Furthermore, both HJ24 and cDNA can regulate the deactivation and activation of PTP. The HJ24 aptamer can define the function of Shp2 phosphatase in normal physiological or under pathological conditions and can be an important ligand for developing new therapeutics for Shp2-dependent cancers and other diseases.

Although most studies state that the antiproliferative effect is caused by binding between the target and G4, during a study concerning two aptamers. Hu et al. reported that this mode of action was not the only one. Both the aptamers S13 (15-mer) and S50 (22-mer) form a parallel G4 structure [91]. Their binding, internalization, and antiproliferative activity in cancer cell lines (A549 cells and MCF10CA1d) and non-cancer cell lines (MCF10A1 and HBE 135-E6E7) were studied and compared with nucleolin-binding AS1411 and TBA. Both aptamers showed good binding properties and internalization in these cell lines. Moreover, these aptamers demonstrated different uptakes. For the S50 aptamer, the uptake was not increased with prolonged incubation time from 1 to 4 h. For the S13 aptamer, the highest cellular uptake was reached around 2 h after incubation, and then decreased with elongation of incubation time from 2 h to 4 h. In this study, only S50 showed strong antiproliferation action against cancer cells. The data suggests that the tumor-selective antiproliferative effect of G-rich oligonucleotides does not directly depend on the binding of the G-rich aptamer to the cells.

In 2003, literature data reported on the usefulness of TBA variants as potential antiproliferative compounds capable of inhibiting the growth of cancer cells [92]. This significant shift in the TBA application resulted in change in the direction of research. Since then, the TBA aptamer has been considered not only as potential anticoagulant agent but also as an antiproliferative compound. The abovementioned properties of TBA were further enhanced by the introduction of a dibenzyl linker [93], inversion of polarity sites [94], 5-hydroxymethyl-2′-deoxyuridine [52], and 4-thiouridine [40]. Furthermore, TBA could reduce the frequency of metastatic events by diminishing thrombin-mediated soluble fibrin formation [95], which operates as a cross-linking moiety that initiates tumor cell capturing and ensures adhesive stability.

## 4. Antiviral Agents

Recently, some drugs based on G-rich aptamers have been approved for antiviral therapy; examples are fomivirsen (antisense oligonucleotide), which is used to treat retinitis caused by cytomegalovirus (CMV) and pegaptanib (Macugen), which targets VEGF and is used to treat neovascular age-related macular degeneration. However, these two therapeutic drugs were withdrawn from clinical practice because of insufficient benefits related to their use [96,97].

However, studies on the therapeutic use of G-rich-aptamers have continued; therefore, these compounds are a major class of aptamers against human immunodeficiency virus (HIV).

The first G4 aptamer against HIV was the ISIS5320, which forms a tetramolecular, parallel G4 structure with a phosphorothioate backbone targeting the gp120 protein [98]. Gp120 is a glycoprotein from the outside layer of the HIV. This protein is essential for virus infectivity and facilitates HIV entry into host cells [99]. The principal domain of the protein is the V3 loop which is essential for virus infection ability. In HIV strains the V3 loop has a high percentage of positively charged amino acids. The higher cationic composition of the V3 loop is related to the virus-mediated cell fusion and rapid viral replication [98]. Moreover, the ISIS5320 aptamer can inhibit the virus adsorption and cell fusion. The G4 structure with the phosphorothioate backbone has a high thioanionic charge density, which is crucial for the antiviral activity. This G4 structure might be the basis for its strong interaction with the cationic V3 loop.

**Figure 3:**
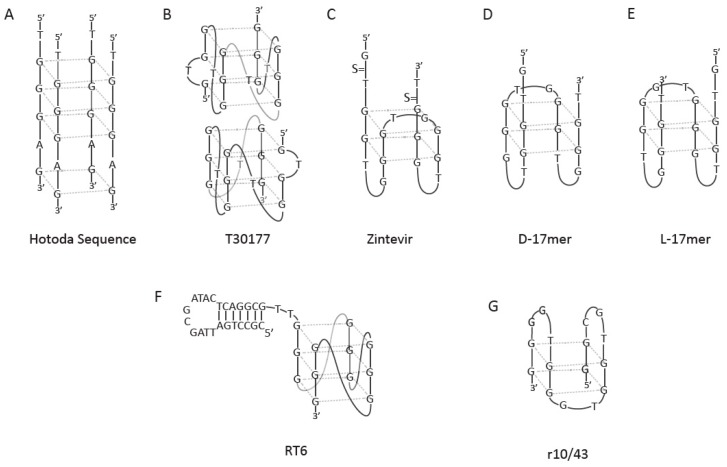
G-quadruplex models for some antiviral agents.

Another potent gp120 inhibitor is Hotoda’s sequence, d(TGGGAG) (Figure 3A), which was the starting point for a series of modifications at either the 5′ or the 3′ end [100]. The presence of abovementioned modifications improved the stability of this tetramolecular parallel G4 structure. It was demonstrated that G4 formation and the presence of large aromatic substituents at the 5′-end were crucial for the antiviral activity. Pedersen et al. also modified this sequence using LNA and (*R*)-1-*O*-[4-(1-pyrenylethynyl)phenylmethyl] glycerol (twisted intercalating nucleic acid, TINA) at both the terminal and internal positions of G4 [101]. The incorporation of LNA and TINA monomers demonstrated an improvement in anti-HIV-1 activity and thermodynamic stability of these modified aptamers. Thus, the above details indicate that a more stable G4 structure might be responsible for the increased antiviral activity.

The HIV attachment to the host cell can be interfered by targeting the nucleolin. The AS1411 aptamer, in addition to its anticancer properties, showed a potent antiviral activity [79]. G4 was able to inhibit HIV-1 replication in a cellular context at 1000-fold lower concentrations of exposure with reference to the efficient anticancer dose. Perrone et al. demonstrated that the AS1411 aptamer had the potential to block HIV-1 infection by binding to NCL on the cell surface and interfering with the HIV-1 cell attachment [102]. Importantly, the aptamer showed high selectivity indexes and no cytotoxic effects under experimental conditions.

Another way to interfere with HIV activity is the application of G-rich aptamers against HIV integrase which is an enzyme that allows the genetic material of HIV to be integrated into the DNA of the infected cell [103]. One example of such compound is the T30177 aptamer, which forms an intramolecular dimeric G4 structure in K^+^ solution. This structure comprises a total of six G-tetrad layers created by the stacking of two propeller-type, parallel-stranded G4 units (Figure 3B). This aptamer, named AR177, was the first HIV integrase inhibitor tested in clinical trials [104]. This aptamer showed high efficacy to inhibit the replication of multiple laboratory strains and clinical isolates of HIV-1; therefore, it had a wide range of therapeutic indices, according to the viral strain and the cell line used in a specified assay. Furthermore, the aptamer was able to suppress HIV-1 activity for more than four weeks after initial treatment in culture, and was stable in serum and in cells [101]. In addition, introduction of phosphate at the 5′-end inhibited the dimerization of this G4 because of a negative charge–charge repulsion. Furthermore, the presence of the 4,4′-dimethoxytrityl group at the 5′-end of the aptamer resulted in a more active analog that showed signs of aggregation and formed the multimeric G4 species in solution.

Zintevir is an analog of T30177, which has a different target than the parental compound, the HIV-1 gp120 envelope protein [105]. Furthermore, the aptamer was partially phosphorothioated at both termini to increase in vivo stability (Figure 3C). To confirm if HIV-1-related proteins recognize non-stereo specifically and if their specific ligands are compatible with other viral proteins, a phosphodiester analog of Zintevir (D-17mer) and its mirror L-17mer were synthetized (Figure 3D,E). The results suggested that the gp120 envelope protein was the target for L-17mer, which exhibited anti-HIV1 activity similar to Zintevir. Based on the above, it can be concluded that the interaction of Zintevir with the gp120 molecule does not depend on its chirality.

In some cases, HIV showed some resistance to the T30177 aptamer [106,107]. In that situation, the 93del aptamer, a derivative of ODN93 (Andevir), can be used. 93del aptamer is a 16-nt dimeric parallel interlocked G4 that has the potential to inhibit HIV integrase [108]. In addition to integrase inhibition activity, 93del can have an inhibitory effect in other steps of viral infection such as reverse transcription and integration.

HIV reverse transcriptase (RT) has been a target for G-rich aptamers. RT is a multifunctional enzyme, essential for virus replication, and is composed of two domains, i.e., the DNA polymerase and the RNase H domains [109]. The RT6 aptamer is the first identified RT inhibitor and has a bimodular structure comprising a 5′-stem-loop module connected at the 3′ to a parallel G4 structure (Figure 3F). This bimodular RT-binding aptamer represents a different architecture of RT antagonists, leading to the possibility of developing different drugs and more studies for HIV-1 replication.

G-rich antiviral aptamers are applied not just as anti-HIV agents. One example is the influenza A virus (IAV) aptamer [110]. This aptamer forms a parallel G4 structure with specificity to protein 1 (NS1) of IAV with the purpose of inhibiting its function. NS1 of IAV inhibits the host’s innate immune response by antagonizing the induction of interferons (IFNs) production. Recently, the selected aptamer was shown to have the capacity to induce IFN-b by suppressing the function of NS1. Notably, this aptamer was able to inhibit viral replication without affecting the cell viability. The blocking of NS1 activity by the aptamer indicates its strong potential for being developed into an antiviral agent against IAV infection.

Another interesting application of antiviral G-rich aptamers is the treatment for hepatovirus A (HAV) infection [111]. The d(G5T) aptamer forms a parallel G4 structure and binds to the HAV 3C protease, interfering with its function and having an inhibitory effect. Enzyme 3C protease plays a central role in the life cycle of HAV; therefore, interfering with this process using the aptamer has a pharmaceutical significance.

The r10/43 aptamer was selected for its ability to bind to the hepatitis C virus (HCV) RNA-dependent RNA polymerase (RdRp) and inhibiting its polymerase activity [112]. This aptamer forms an antiparallel G4 structure (Figure 3G); moreover, the r10/43 aptamer exerted an inhibitory effect on norovirus and φ6 polymerase. Results suggest that the r10/43 aptamer inhibited the polymerase in a competitive way. The r10/43 aptamer binds to or in close proximity to the template-binding region and blocks template access for the polymerase, and consequently blocks HCV replication.

Recently, the discovery of interleukin-6-receptor (IL-6R) aptamers, which are referent to rAID-1, and AIR-3A aptamers, was reported. These two aptamers form a parallel-stranded G4-structure and bind to IL-6R and HIV-1 integrase [113]. They are able to inhibit the HIV-1 integrase 3′ processing activity *in vitro*. Moreover, these aptamers are able to prevent HIV de novo infection with same efficiency as the well-known HIV-1 integrase inhibitor T30175. Magbanua et al. reported that both interactions of the aptamers with the targets are dependent on G4 structure formation.

## 5. Aptasensors

In the past few years, the role of G-rich aptamers has been increasingly studied to mediate analytic detection, in a system called aptasensors. The sensing procedure of aptasensors is similar to that of other biosensors, i.e., they recognize the target and transfer the output signal. [114]. This signal is recognized and the information is measurable. Moreover, depending on the mechanism of action, aptasensors can be divided in three main groups such as electrochemical, optical, and acoustic aptasensors. They also have some advantages compared to normal biosensors, i.e., high affinity, stability, and ease of regeneration. Their 3D folding capability after binding allows for simple and direct target detection.

Here, we discuss some recent examples of different applications of this type of system and the latest achievements in the field.

**Figure 4:**
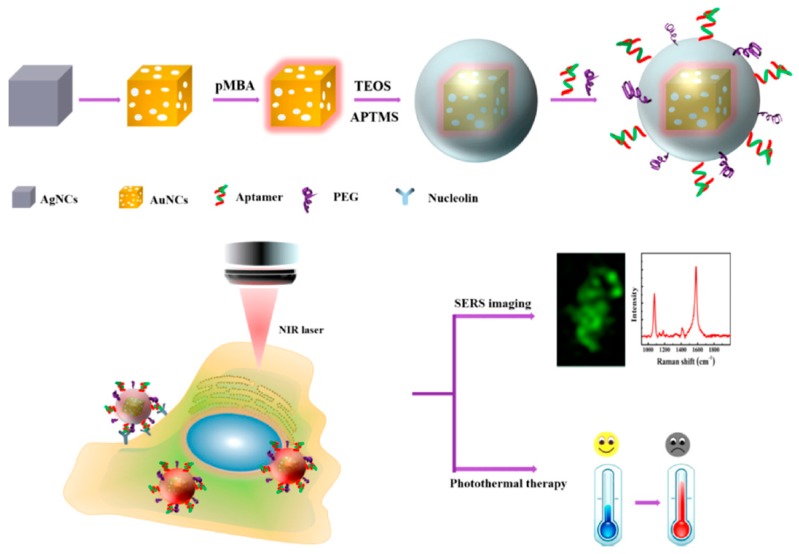
Schematic preparation of fluorescent aptasensor system for Cu^2+^ detection (top) and its application using SERS-imaging and photothermal therapy (bottom) [117]. pMBA: 4- mercaptobenzoic acid, TEOS: tetraethylorthosilicate, APTMS: (3-aminopropyl)trimethoxysilane.

The AS1411 aptamer, in addition to its anticancer and antiviral activity, is used for developing aptasensors. This aptamer can be applied as a fluorescent aptasensor for Cu^2+^ detection characterized by high affinity and specificity. Copper is an essential element for diverse processes in biological systems such as oxidative stress protection, cell growth, and development, it helps to heal injured human skin tissues and is involved in pigmentation [115]. The established aptasensor was applied to diagnose diseases such as Alzheimer’s disease, Wilson’s disease, and diabetes [116]. The mechanism of this fluorescent aptasensor is based on the selective recognition of Cu^2+^ that binds to AS1411 on the surface of magnetic beads, hindering the hybridization of AS1411 with complementary strand and resulting in a weak fluorescence. On the contrary, in the absence of Cu^2+^, AS1411 can bind to its complementary strand, generating a high fluorescent signal upon the addition of gel red. It was demonstrated that the aptasensor could efficiently differentiate between patients suffering from these diseases and healthy people based on their serum Cu^2+^ levels. This confirmed that the developed system could be applied as a novel diagnostic tool for these diseases. The nanoprobe, formed by an Au nanocage coated with SiO_2_ (AuNC/ SiO_2_), uses the AS1411 aptamer as a bifunctional theranostic platform for cellular surface-enhanced Raman scattering (SERS) imaging and NIR-triggered photothermal therapy (Figure 4). The conjugate of AS1411, named AuNC/SiO_2_/Apt, has the capacity of providing a higher SERS enhancement and sufficient hyperthermia because of the plasmon effects and hollow core nanostructure [117]. Moreover, the nanoprobe shows good photostability and biocompatibility through photothermal treatment because of the presence of an inert SiO_2_ shell. These unique traits make the current AuNC/SiO_2_/Apt nanoprobes promising theranostic agents for both cellular SERS mapping and photothermal therapy. They can recognize MCF-7 cells with nucleolin-overexpression through SERS imaging. Furthermore, the nanoprobes are characterized by sufficient therapeutic outcome upon NIR laser irradiation at a relatively low power density. AuNC/SiO_2_/Apt nanoprobes have considerable potential to be applied for tracing tumor sites and biodistribution in vivo, as well as SERS imaging-guided diagnosing and remote-controlled photothermal annihilation of cancer cells.

**Figure 5:**
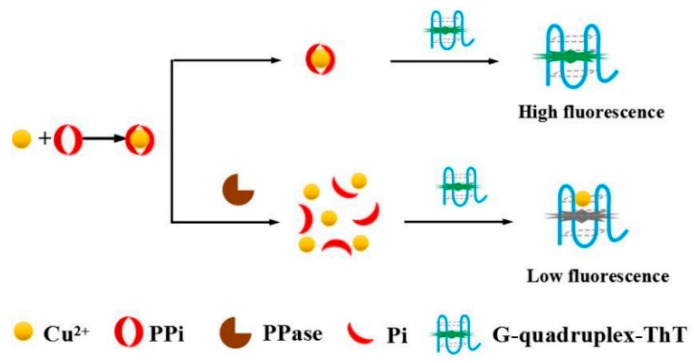
Fluorometric approach to quantify the based on thioflavin T dye [118].

A G-rich thioflavin T aptamer is proposed to be used in a fluorometric approach for detecting inorganic pyrophosphatase (PPase) [118]. In the catalysis of PPase, the inorganic pyrophosphate (PPi) molecule is converted into two orthophosphate (Pi) ions. PPase is associated with several clinical diseases and biological processes. Some of these processes include phosphorus and carbohydrate metabolism, calcium absorption, bone formation, lipid synthesis and degradation, and DNA synthesis. Moreover, PPase plays a significant role in energy anabolism that occurs in all organisms and results in cell death. The mechanism of detection is based on fluorescent response induced by G4 structure transformation which is induced by the presence of Cu^2+^ ions (Figure 5). Once PPase is present, the Cu^2+^ stays free because of the PPase-catalyzed hydrolysis of PPi to inorganic phosphate (Pi). The release of Cu^2+^ leads to interactions with ThT and unfolding of G4 structure, resulting in fluorescence quenching. In the absence of PPase, Cu^2+^ can form a Cu^2+/^PPi complex with pyrophosphate. When this complex is present, thioflavin T (ThT) mediated G4 structure formation produces a fluorescent signal. This simplistic and quantitative strategy can have potential applications in PPase-related clinical diagnosis, analysis, and functional studies.

**Figure 6:**
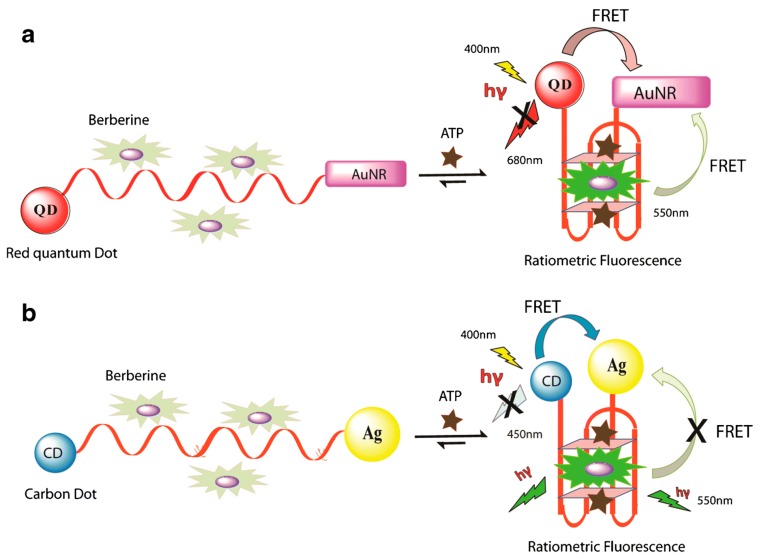
The Forster resonance energy transfer (FRET)-based ATP aptasensors. (**a**) ATP-binding oligonucleotide labeled with red quantum dots (RQDs) and gold nanorods (AuNRs), which in the presence of ATP and berberine, folds into a G4 structure. FRET process results in the quenching of RQD fluorescence and an increase of berberine fluorescence is simultaneously observed. (**b**) Ag- and CD-labeled ATP-binding oligonucleotide system forming the G4 structure in the presence of ATP and berberine [119].

Furthermore, based on two fluorescent aptasensors, two luminescent methods were developed for detecting ATP in biochemical systems [119]. The first method is a label-free fluorescent turn-on method using a G-rich ATP aptamer sequence and the DNA-binding agent berberine complex. The ATP preferentially binds with the aptamer and induces conformational changes from the single-stranded oligonucleotide into a G4 structure. Moreover, the association of berberine with G4 results in improvement of the fluorescence signal. The second method uses the ratiometric fluorescent method based on Forster resonance energy transfer (FRET) for detecting ATP. This system comprises berberine, which is the ATP-binding aptamer labeled with gold nanorods (AuNRs) as a quencher part and red quantum dots (RQD, donor) or carbon dots (CDs) as fluorophore at the 5′ and 3′ terminal, respectively. After adding ATP and berberine, ATP specifically binds to the aptamer, leading to the formation of G4, followed by the interaction of berberine with G4. As a result, an improvement of fluorescence of berberine is observed, while that of RQD and CDs is significantly quenched via the FRET process (Figure 6). These two techniques, based on label-free and fluorescently labeled aptasensors for detecting ATP in the presence of the G4-binding agent, have the potential to detect a variety of small molecules, more sensitively and selectively than others aptasensors.

Recently, two aptamers, R1.2 and R1.3 used for constructing effective aptamer-based biosensors for the detection of cancer-relevant biomarkers were described. These are demonstrated to be very polymorphic and fold into stable unimolecular G4 structure in K^+^ buffer [120]. Both aptamers can exclusively bind to the membrane-bound immunoglobulin M (mIgM) expressed on B cells lymphoma in the presence of K^+^. The G4 formation is favored and essential for binding. These aptamers represent a new class of aptasensors for detecting cancer-relevant biomarkers.

Existing detection systems can be improved, similar to the system applied for the VEGF protein [121]. VEGF is a protein that acts as a key regulator in angiogenesis and plays a role in initiating tumor angiogenesis. This protein is also an important biomarker for age-related macular degeneration. Previous studies have reported that the VEap12 aptamer recognizes the VEGF protein. It is noteworthy that the VEap12 aptamer folds into a G4 structure, which is important for VEGF recognition because it recognizes the receptor-binding domain and the heparin-binding domain. This aptamer was improved by in silico maturation which led to the bivalent 3R02 aptamer and showed better results with VEGF recognition [122]. Both VEap12 and dimer 3R02 formed an antiparallel G4 structure. Dimer 3R02 contained two independent monomeric 3R02 structures and a 10-mer thymine linker (T10). Nonaka et al. built a VEGF detection system using a VEGF antibody as the capture molecule and monovalent 3R02 as the detection molecule. This aptamer has better affinity to VEGF and is a better alternative for developing more sensitive detection systems compared with the system based on VEap121.

**Figure 7:**
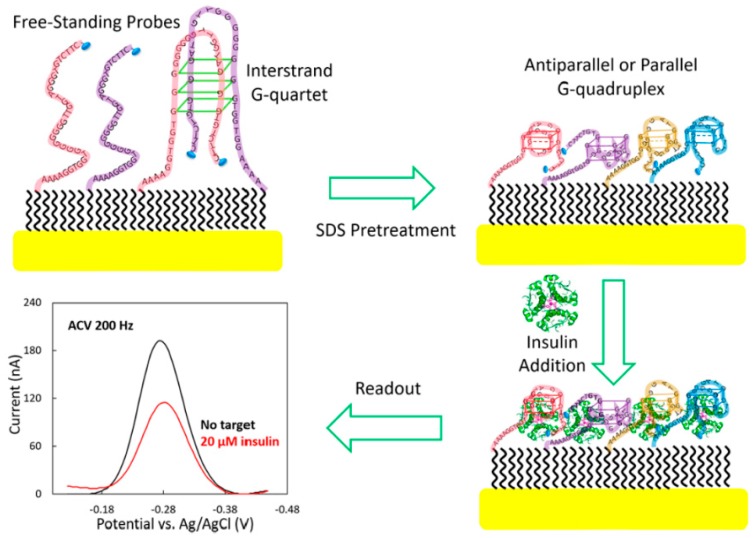
Representation of the electrochemical sensor based on IGA3 aptamer (E-AB) for the detection of insulin levels [21].

A remarkable aptamer-based electrochemical sensor (E-AB) was developed for monitoring insulin levels in real-time. This sensor is based in the G-rich IGA3 aptamer, first selected and reported by Yoshida et al., which folds into a G4 on recognition of insulin [123]. The signaling of this new sensor is based on the binding-induced steric hindrance of electron transfer between the redox label and electrode surface (Figure 7) [21]. To achieve reproducibility of E-AB insulin sensors, 10% SDS pretreatment is required to avoid the formation of interstrand G-quartets. The insulin-end sensor with a methylene blue (MB) label at the proximal end of the IGA3 aptamer probe sequence is more advantageous than its counterpart with an MB label modification located in the middle of the IGA3 aptamer probe sequence. This sensor offers an LOD (limit of detection) down to 20 nM and is rapid as 90% signal saturation is achieved within 60 s. The E-AB insulin sensor shows rapid kinetics, and is sensitive, specific, and selective, demonstrating that this sensor can be used for real-time monitoring of insulin.

**Figure 8:**
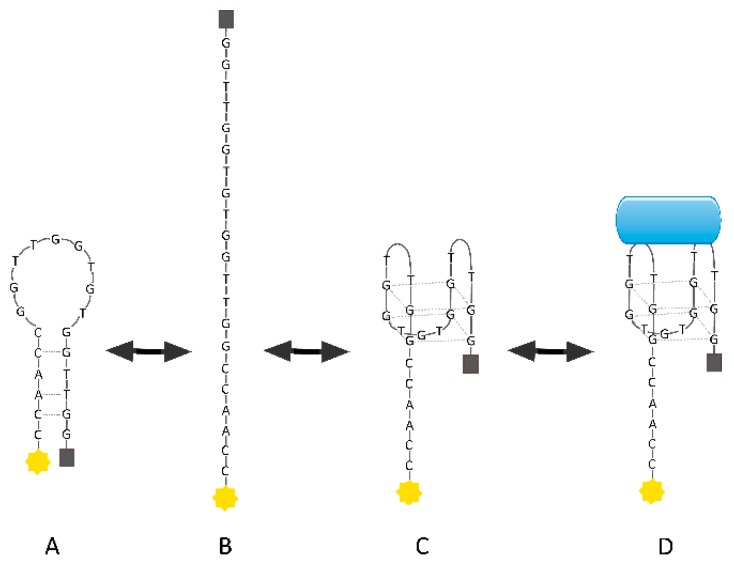
Mechanism of thrombin aptamer beacon action: (**a**) Aptamer beacon in quenched stem-loop conformation, (**b**) unfolded conformation, (**c**) aptamer in G-quartet conformation, and (**d**) aptamer beacon with bound thrombin [125].

TBA was successfully used in aptasensors because of its high specificity and ability to bind to the thrombin molecule even at low concentrations. In 2000, the first fiber-optic biosensor for measuring thrombin concentration with a detection limit of 1 nM was developed [124]. In this system, TBA was immobilized on the surface of silica microspheres and placed in microwells at the distal tip of an imaging fiber. The thrombin used in this experiment was fluorescently labeled and signal detection was performed using a modified epifluorescence microscope system. An equally interesting approach was presented by Santon et al. who designed a new class of molecules termed aptamer beacons that were used for thrombin detection in solution [125]. These compounds consisted of a TBA sequence with additional nucleotides at the 5′-end, complementary to nucleotides at the 3′-end of the aptamer. Moreover, the fluorophore and quencher were attached to 5′- and 3′-ends, respectively. The binding of thrombin to the aptamer beacon resulted in conformational change and increase in the distance between the fluorophore and quencher (Figure 8). Any signal emission occurring in this situation could be correlated with the protein amount.

**Figure 9:**
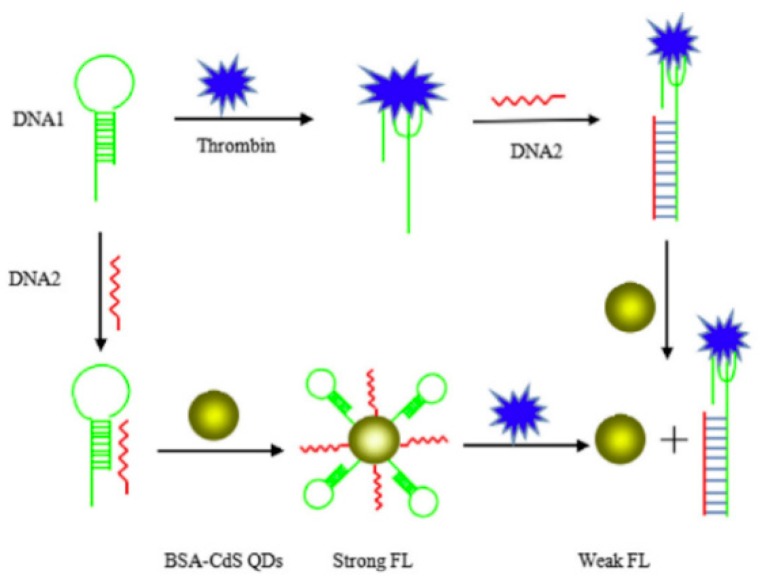
Fluorescent detection of thrombin based on BSA-Cds QDs [126].

A similar strategy was applied in the aptasensor designed by Na et al. [126]. The system consisted of quantum dots combined with bovine serum albumin (BSA-QD) and two oligonucleotides: oligomer DNA1 possessing hairpin structure and TBA sequence and complementary to its stem fragment oligomer DNA2. Oligomer DNA2 bound BSA-QD via electrostatic interactions, which resulted in the emission of a fluorescent signal. The presence of thrombin in the solution triggered change in the oligomer DNA1 structure and facilitated its hybridization with oligomer DNA2 (Figure 9). The resulting complex lost its ability to interact with quantum dots and led to the disappearance of the fluorescent signal, which was inversely proportional to the amount of thrombin in the solution.

Furthermore, atomic force microscopy (AFM) [127], surface plasmon resonance spectroscopy (SPR) [128], and Raman spectroscopy [129] were used as alternative detection methods. Signal amplification was achieved by using gold nanoparticles [130], silver [129,131,132], carbon nanotubes [133], graphene [134,135], and enzymatic reactions [130].

**Figure 10:**
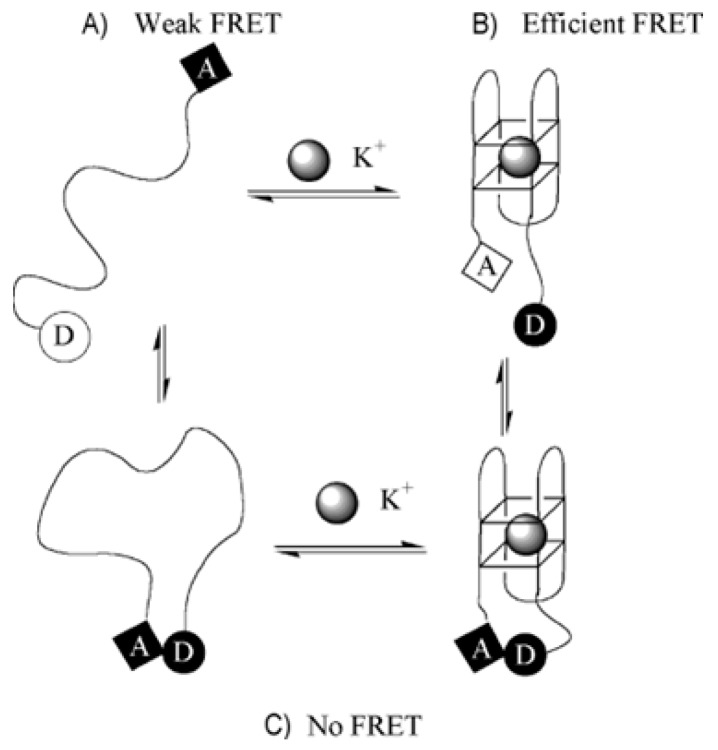
The model of action of aptasensor based on TBA conjugated with fluorophores: 6-carboxytetramethylrhodamine (TAMRA) (acceptor, A) and 6-carboxyfluorescein (FAM) (donor, D). FRET occurs when the distance between D and A is sufficient– (**A**) and (**B**). When the G4 is formed because of the presence of K^+^, the phenomenon is not observed (**C**) [136].

The conformational changes of TBA induced by the presence of potassium were exploited to develop sensors for monitoring ion concentration in living organisms. Nagatoishi et al. synthesized TBA conjugated with fluorophores at its both ends: 6-carboxytetramethylrhodamine (TAMRA) and 6-carboxyfluorescein (FAM) [136]. When potassium ions were present in the environment, TBA folded into a G4 structure, which provided an optimal distance between the donor and acceptor and enabled the FRET phenomenon to occur (Figure 10). However, the above-mentioned system had some limitations. Moreover, a false positive outcome was observed under the same conditions, which could be a result of interaction between the donor and acceptor from two different molecules.

**Figure 11:**
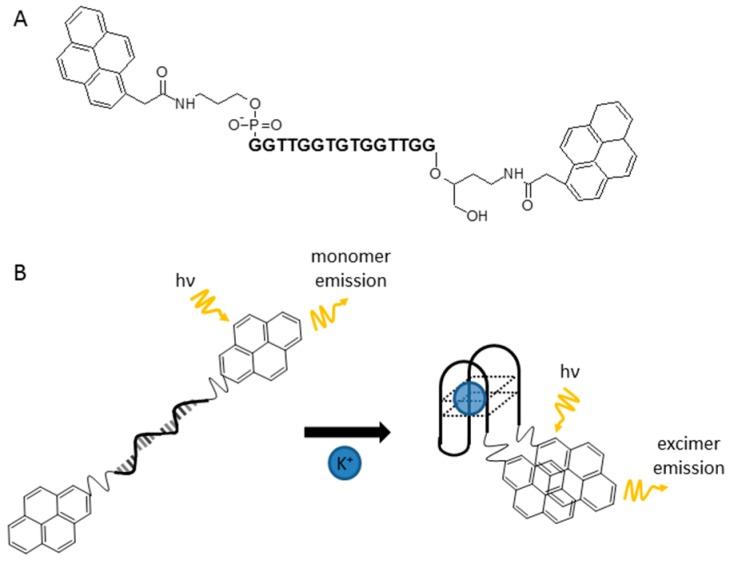
The chemical structure of potassium-sensing oligonucleotide (**a**) and the mechanism of K^+^ detection (**b**) [137].

Takenaka et al. managed to avoid this problem by placing pyrene residues at each aptamer termini [137]. These compounds, because of the formation of G4 induced by the presence of potassium ions, were arranged in a specific configuration and formed excimers in the excited state (Figure 11). This resulted in the appearance of a strong band in the long-range fluorescence spectrum.

## 6. Other Applications

G-rich aptamers can be used for other therapeutic purposes in addition to anticancer, anticoagulant, or antiviral treatment. Other applications, such as the PPK2 G9 aptamer targeting *Mycobacterium tuberculosis* (Mtb), have already been reported [138]. This aptamer forms an antiparallel G4 structure and is targeted toward polyphosphate kinase (PPK) protein families. The PPK protein families regulate the inorganic polyphosphate (polyP) intracellular metabolism, and inhibition of the PPK activity is a potential approach to disrupt polyP-dependent processes in pathogenic organisms. The PPK2 G9 aptamer was demonstrated to bind strongly to the protein and efficiently inhibit its polyphosphate-dependent nucleoside diphosphate kinase (NDK) activities, resulting in Mtb inhibition. The other examples of aptamers having therapeutic applications against Mtb are HupB-4T and HupB-13T, both of which form a parallel G4 structure [139]. They demonstrated high stability, affinity, and selectivity for HupB protein. The HupB protein is a DNA^−^binding protein HU-beta, which facilitates mycobacteria entry into host cells and regulates iron homeostasis, an important function for Mtb survival in intracellular infection. These aptamers inhibit the functions of the Mtb HupB protein, thus demonstrating great potential for being developed into biocompatible inhibitors of Mtb survival.

**Figure 12:**
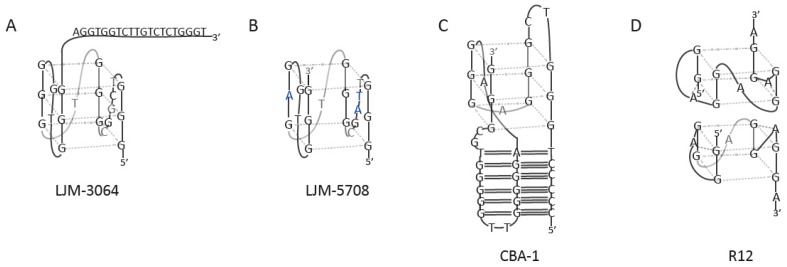
G-quadruplex models of aptamers targeted toward myelin (**A** and **B**), codeine (**C**) and prion protein (**D**).

In addition to being good candidates as antibacterial agents, aptamers could be successfully used for treating multiple sclerosis (MS), which is a disease that causes demyelination of the central nervous system and affects ~0.1% of the global population [19]. In relation to MS, the action of aptamer LJM-3064, which is a 40mer, with a 5′ G4-forming half and an unstructured 3′ half (Figure 12A) was described [140]. This aptamer forms an antiparallel G4 structure with intramolecular interaction in the presence of Na^+^; however, when small amounts of K^+^ are present (similar composition of fluid and plasma blood), the structure changes to a parallel G4 structure with intramolecular interaction. This aptamer has higher affinity to bind to myelin and was capable of stimulating remyelination in the mouse model. The following studies were performed by the same research group with LJM-5708, a 20-nt variant of the previous aptamer, forming a stable parallel G4 structure (Figure 12B) [19]. LJM-5708 demonstrated enhanced myelin binding and was able to bind to human oligodendroglioma *in vitro*. Furthermore, the aptamer conjugated with streptavidin, avidin, and neutravidin, and retained its activity. Therefore, LJM-5708 became a strong candidate for future preclinical testing such as pharmacokinetic analysis and remyelination aptamer technologies.

G-rich aptamers have been used to treat skeletal diseases. There are many diseases that can affect the skeleton such as osteomyelitis and osteoporosis. The Scl2 aptamer was reported to improve the treatment for skeletal diseases [141]. This aptamer has a parallel G4 structure with high affinity for sclerostin. This extracellular protein, secreted by osteocytes, negatively regulates bone formation. The Scl2 aptamer potentially inhibits the antagonistic effect of sclerostin on Wnt signaling; however, targeting Wnt signaling can be problematic because it is not an on-off process. The constant activation of Wnt signaling can result in cancer; however, its complete inhibition can lead to occurrence of osteoporosis and heart failure. One possible way to avoid these two events and control anti-sclerostin activity is by using aptamer antidotes based on complementary nucleotide base pairing. Some examples of aptamer antidotes already exist, such as an antidote to reverse anticoagulation caused by Ch-9.3t (an anticoagulant aptamer) [142] or the universal antidotes based on protein and polymers [143], which are able to capture oligonucleotides and reverse their activity [141].

In addition to aptamers with antibacterial activity, aptamers may also have antifungal applications. Fungal infections have been increasing over the past few years. Once the host’s defenses are decreased, *Saccharomyces cerevisiae* (*S. cerevisiae*) becomes an opportunist fungal pathogen and causes severe health problems. One aptamer with antifungal properties is the GVEGF aptamer [144]. This aptamer was applied to *S. cerevisiae* to inhibit the chitin synthesis pathway, an essential process for fungi cell growth. Normally, the VEGF aptamer forms an antiparallel G4 structure; however, when it is modified with LNA, a single parallel G4 structure, named GVEGF aptamer, is observed [145]. This aptamer variant demonstrated high thermal stability, high nuclease resistance, and improved affinity to the CHS6 protein. This binding was responsible for interrupting the CHS5-CHS6 complex formation. It is noteworthy that the CHS3 protein is responsible for chitin production and is essential for cellular growth. This protein activity requires the assembly of a protein complex, i.e., the CHS5-CHS6 complex. The binding of the GVEGF aptamer to the CHS6 protein disrupts the CHS5-CHS6 complex formation and affects the CHS3 protein transportation to the lipid membrane and consequently disorders chitin localization. The GVEGF aptamer was shown to be a promising antifungal agent for drug development [144].

It is noteworthy that G-rich aptamers can be applied against inflammatory diseases. A good example is the VR11 aptamer, which binds to human tumor necrosis factor-alpha (TNFα), a crucial component of the cytokine network linked to inflammatory diseases [146]. The G-rich content of the VR11 sequence displays an 18-nucleotide-long stretch and contains four GG repeats that may stabilize the aptamer and its intramolecular G4 structure, which supports association with TNFα. The VR11 aptamer, which inhibited TNFα signaling, was able to prevent TNFα-induced apoptosis and reduced nitric oxide (NO) production in cultured cells for up to 24 h. Thus, VR11 may represent a simpler, synthetic scaffold than the antibodies or protein domains, and could serve as a non-immunogenic oligonucleotide-based inhibitor of TNFα.

Recently, a study was conducted on the codeine binding aptamer (CBA-0) and its derivative CBA-1 [147], both of which are G-rich DNA sequences with the original CBA-0 aptamer containing six G-tracts and a C-tract (5C-T-3G-TC-3G-A-3G-AA-5G-TT-5G-TGC-2G). The CBA-0 structure comprises the G4 formed by four of the G-tracts and a G·GC triplex created by a 5C-tract and two 5G-tracts. The derivative CBA-1 was achieved by adding a G-base to the 3′-end of CBA-0. This aptamer folded into the parallel G4 structure with four 3G-tracts (Figure 12C). It is noteworthy that the junction formed by the triplex and the G4 of CBA-1 creates a cavity where the codeine binds. Codeine can be observed only when both structures are connected and form a well-defined triplex-quadruplex scaffold. This scaffold can be implicated in the rational design of aptameric sensors because of the formation of a unique structure when binding to codeine. Moreover, it has the potential to function as a regulator of gene expression, thus offering new perceptions into DNA architecture and molecular recognition.

The existence of the RNA G-quadruplex in the genome has been known for a long time; moreover, it is essential for many biological functions and processes. The development of SELEX technology makes it possible to create new RNA G4 aptamers for different applications and therapeutics [148]. One of the most well-recognized examples of G4-forming RNA aptamers is the R12 aptamer associated with prion disease prevention [148,149]. This aptamer forms an intramolecular, parallel G4 structure with two G-quartet layers. The top G-quartet is further stabilized by two additional adenine residues from the loops (Figure 12D) [150]. This aptamer forms a dimer when each monomer simultaneously binds to two portions of the N-terminal half of the abnormal form of the prion protein (PrP^sc^). Prion proteins (PrPs) are physiologically found most abundantly in the brain; however, the abnormal folding of the PrPs leads to brain damage and causes pathogenesis such as the bovine spongiform encephalopathy (BSE). Being a good candidate for medicine against BSE, the binding of the R12 aptamer with PrPs reduces the conversion to PrP^sc^, resulting in anti-prion activity in mouse neuronal cells. These discoveries could be used to improve RNA aptamer-based drugs against prion and Alzheimer’s diseases.

RNA-G4 based aptamers can act as modulators in the pathological response to imbalance metabolites. They can be effective ligands for small molecular weight compounds, acting as physiological effectors or mediators. Lévesque et al. demonstrated that RNA aptamers can specifically bind to thyroid hormones, which are suitable metabolites for a potential human riboswitch. These RNA structures can include a G-rich motif, with a G4 structure, adjacent to a helical region [151]. They showed that the presence of thyroxine was important for forming the parallel-stranded G4 structure. Furthermore, the binding is shown to be specific to thyroxine (T4) and triiodothyronine (T3), the active forms of the hormone; however, other inactive derivatives, including thyronine (T0), do not support G4 formation.

## 7. Conclusions

After the development of SELEX technology in 1990 and with subsequent improvements in various aspects, aptamers have been extensively studied. Over time, G-rich aptamers have been shown to have several advantages in numerous fields. Because of their interesting properties, including the stability of their structure, improvement of the electrostatic interactions, high accessibility to chemical modifications, and low synthetic cost or facile manipulation, researchers can develop and improve personalized treatments, medicinal drugs, detection methods, imaging, among various applications.

In a certain manner, G-rich aptamers have demonstrated some advantages over monoclonal antibodies, such as simple scaffold, smaller size, no immunogenicity, reversibility of action, and ease of manufacture and storage. G-quadruplexes have shown to be strong and useful alternatives to antibodies in targeted therapy, as well as for in vitro and in vivo diagnosis or biomarker detection.

Throughout this review, we showed that the unique folding characteristics of G4 allow for the recognition of a wide range of molecular targets such as proteins, viruses, and bacteria. In the majority of examples, the disruption of the G4 structure leads to the loss of biological activity of G-rich aptamers. Therefore, the preference of molecules to adopt the G4 structure appears to be essential for their biological function and interactions with the target. Moreover, their versatility and plasticity in forming these structures and binding to a variety of molecular targets have proven indispensable for many detection methods with increased selectivity and sensitivity.

An interesting feature of G-rich structures is that the same G-rich aptamer, such as the AS1411 aptamer, can be applied in different systems with direct therapeutic potential; moreover, it can be used as an aptasensor or drug carrier to facilitate specific cellular recognition and uptake. Furthermore, more than one biological effect can be observed for single aptamer, such as TBA, which is a potent anticoagulant agent and, under specific conditions, can be characterized by reasonable antiproliferative activity. Although G-rich aptamers have a wide range of applications, the molecules still face some obstacles such as nuclease degradation or renal excretion.

Therefore, additional studies are required, particularly at the preclinical and clinical levels to make G-rich aptamers widely used in the future, especially in therapeutic and diagnostic fields. Notably, most of the recent research is based on DNA G-rich aptamers. However, since RNA G-rich aptamers are involved in many biological processes, the increased focus on these aptamers could constitute interesting alternatives for currently applied biomedical approaches.
